# Expression and Transformation Characteristics of a Novel Glutamic Acid Decarboxylase LcGAD10s and Its Application on Sufu Processing

**DOI:** 10.3390/foods12173186

**Published:** 2023-08-24

**Authors:** Zhou Chen, Run Wang, Yanyin Song, Aijin Ma, Siting Li, Yingmin Jia

**Affiliations:** School of Food and Health, Beijing Technology and Business University, Beijing 100048, China; zhouch2017@btbu.edu.cn (Z.C.); 2130022102@st.btbu.edu.cn (R.W.); songyanyin0214@163.com (Y.S.); 20190601@btbu.edu.cn (A.M.); lisiting@btbu.edu.cn (S.L.)

**Keywords:** gamma-aminobutyric acid, glutamate decarboxylase, *Latilactobacillus curvatus*, expression, characterization, sufu

## Abstract

Gamma-aminobutyric acid (GABA) is an important non-proteinogenic amino acid and a potent bioactive compound with many anti-hypertensive and anti-depressant activities. The bioconversion of GABA by glutamic acid decarboxylase (GAD) has been eagerly studied. Herein, novel pyridoxal-5-phosphate monohydrates (PLP)-dependent GAD, which is not quite similar to reporting, was cloned from *Latilactobacillus curvatus* and efficiently expressed in *E. coli*. The conveniently purified GAD (designated LcGAD10s) appeared as a single protein on SDS-PAGE with a molecular mass of 52.0 kDa. LcGAD10s exhibited a specific activity of 303.7 U/mg after purification by Ni–IDA affinity chromatography, with optimal activity at 55 °C and pH 5. LcGAD10s displayed excellent temperature (50 °C) and pH (4–8) stability which relative activity above 80% and 70%, respectively. The enzymatic activity was, respectively, increased and depressed by 130%, and 24% in the presence of Mn^+^ and Cu^2+^. Enzyme activity over 90% can be achieved by adding at least 25 mM of PLP. LcGAD10s was able to efficiently transform 15 g/L GABA with a single-factor optimized reaction of pH (5), temperature (50 °C), time (2 h), LcGAD10s dosage (0.4 U) and monosodium glutamate level (5 g/L). Additionally, LcGAD10s can be applied to a tofu fermentation system to achieve GABA conversion and achieved 14.9 mg/g of GABA conversion when added at 2 U/mL, which is higher than most of the commercial sufu and previous application reports, increasing its functional substances.

## 1. Introduction

γ-Aminobutyric acid (GABA) is a non-protein amino acid that is widely found in nature [1]. As a functional factor, GABA has been proven to be safe for consumption. It has been widely studied for its various physiological functions such as reducing stress and aiding sleep [2], and oral consumption of GABA-enrich foods has been shown to have antihypertensive and antidepressant properties [3]. GABA is gradually being used in food, medicine, agriculture, and animal husbandry, and the development of functional foods containing high concentrations of GABA is being actively pursued [4].

In order to meet the growing commercial demand, there is a clear need to develop more efficient and environmentally friendly processes for the low-cost synthesis of GABA. Among the various production methods, the bioconversion method, including enzymatic method and microbial production, has received considerable focus to satisfy the stringent requirements for an additive in the food, pharmaceutical, and livestock industries [5]. Bioconversion is based on L-glutamic acid (Glu) or monosodium glutamate (MSG) as raw material and glutamic acid decarboxylase as a catalyst, which can produce GABA with higher titer and higher reaction rate [5]. In comparison, the bioconversion method for GABA production is not only efficient and economical but also safer, environmentally friendly and large scale [6]. Therefore, the use of microbial and enzymatic methods to produce GABA has become a hotspot.

Glutamate decarboxylase (GAD; EC 4.1.1.15) is a key enzyme that catalyzes the irreversible α-decarboxylation of L-glutamate (Glu) to GABA [7]. The enzymatic reaction is a more promising approach for the synthesis of GABA through GADs [4]. To date, various GADs have been identified and characterized by several microorganisms [8]. Among them, lactic acid bacteria (LAB) species have a long history of human use, including being recognized as “Generally Recognized as Safe” or “Qualified Presumption of Safety” by the Food and Drug Administration and the European Food Safety Authority, respectively [9]. According to previous reports, various GADs including those derived from *Lactobacillus* [10,11,12] have been expressed and identified from LAB. These LAB have significant GABA titers and often possess highly active endogenous GAD and their beneficial properties have been utilized in fermented foods [5], specially catalyzed by GAD to produce up to 70 g/L of GABA [13]. In summary, LAB can be considered an ideal source of microbial genes encoding GAD, more recent reports have been on heterologous expression and functional characterization of LAB-derived GADs from *Lactobacillus brevi* [14], *Lactobacillus fermentum* [15], *Lactobacillus plantarum* [16], *Lactococcus garvieae* [6] and *L. sakei* [17].

Currently, due to the rapid development of bioinformatics, databases enable rapid screening of target protein sequences for cloning and expression of chemically synthesized enzymes by genetic engineering methods. Therefore, in this study, a hypothetical protein regarded as a novel GAD, LcGAD10s, from *Latilactobacillus curvatus* was expressed in *E. coli*, which has never been reported before, was identified, purified and characterized. In addition, the biochemical properties and the ability of LcGAD10s to transform GABA were further investigated. Moreover, we conduct a preliminary study on the application of LcGAD10s to sufu, for which the application characteristics of LcGAD10s still need to be fully investigated in the future.

## 2. Materials and Methods

### 2.1. Materials

Monosodium glutamate (MSG), PLP, boric acid, phenol and sodium hypochlorite (Solarbio Science & Technology Co., Ltd., Beijing, China). Lowry kit and Isopropyl β-D-Thiogalactoside (IPTG) were purchased from Beyotime (Shanghai, China). PageRuler Prestained Protein Ladder (Thermo Fisher Scientific Inc., Waltham, MA, USA). Chelating Sepharose (Ni-IDA) resin matrix was from GE Life Sciences (Pittsburgh, PA, USA). All the other reagents used were analytical or chromatographic grade unless otherwise noted.

### 2.2. Gene Mining and Cloning of Novel GADs

The BLAST (Basic Local Alignment Search Tool) analysis tool was used to search in the NCBI database (Available online: https://www.ncbi.nlm.nih.gov/, accessed on 1 July 2023), and a hypothetical GAD protein (GenBank No: QWS69943.1) derived from a *Latilactobacillus curvatus* genomic information source, which not identified by expression was identified from the search results. After enzyme site analysis of the target gene and the polyclonal site of the expression plasmid, LcGAD10s was synthesized by adding the appropriate enzyme sites to the upstream and downstream sequences of the target gene using the relevant software. PCR amplification of the gene fragment was performed using *NdeI* and *XhoI* sites (underlined) LcGAD10s-up (ATTCTACATATGATGACTGGCTTCACTCCGGAT) and LcGAD10s-down (ATTCCGCTCGAGCTACTCCAGTCCGATGGACTTCAT). The PCR products were digested by *NdeI* and *XhoI* and subcloned into the pet-28a(+) vector. The recombinant plasmids used were also sequenced and validated. Furthermore, the theoretical isoelectric points (*p*I) of these potential novel GADs were predicted using the ProtParam tool (Available online: https://web.expasy.org/protparam/, accessed on 1 July 2023).

### 2.3. Expression and Purification of the LcGAD10s

LcGAD10s were inoculated into 10 mL of Luria-Bertani (LB) medium (5 g/L yeast paste, 10 g/L tryptophan and 10 g/L NaCl) in a 50 mL flask at 37 °C at 200 rpm. Incubate 2 mL of the overnight seed culture diluted in 100 mL LB medium in a 250 mL flask. LcGAD10s expression was induced by the addition of 1 mM IPTG when the 600 nm optical density reached 0.6. All cultures were supplemented with kanamycin maintenance plasmids fed at a final mass concentration of 50 mg/mL. After 12 h of incubation at 180 rpm at 30 °C, the fermentation broth was collected by centrifugation at 4 °C at 8000 rpm to collect the expression organisms and then resuspended in 0.05 mM Tris-HCl buffer (pH 7.4). After ultrasonic treatment of the cells at 4 °C in an ice bath, the lysed debris was removed by centrifugation at 12,000 rpm for 30 min at 4 °C to remove cell debris, and soluble GAD (crude enzyme) was obtained from the supernatant [18,19].

The supernatant was loaded on a Ni-IDA column (0.8 × 10 cm) filled with Sepharose (Ni-IDA) resin matrix and washed with 20 mM imidazole in the lysis buffer. The GAD was finally eluted using ÄKTA Explorer 100 chromatography system (GE Healthcare, Uppsala, Sweden) by gradually increasing the imidazole concentration (20–300 mM) of the elution buffer. The fractions containing target proteins were collected. The purified enzymes were desalted by ultrafiltration using 10 kDa dialysis bags (EMD Millipore, Billerica, MA, USA) against Na_2_HCO_3_-citric acid buffer (50 mM, pH 4.5), and then stored in the refrigerator at 4 °C [8].

### 2.4. SDS-PAGE Analysis of LcGAD10s

The purified protein was analyzed by 10% (*w/v*) sodium dodecyl sulfate-polyacrylamide gel electrophoresis (SDS-PAGE) for molecular weight size and purity [20]. The molecular mass of the enzyme subunit was determined using a low molecular mass standard (Biodee, Beijing, China) containing rabbit phosphorylase B (97.4 kDa), bovine serum albumin (66.2 kDa), rabbit actin (43.0 kDa), bovine carbonic anhydrase (31.0 kDa), trypsin inhibitor (20.1 kDa) and hen egg white lysozyme (14.4 kDa). 

### 2.5. Enzyme Assay and Protein Determination

The catalytic activity of glutamate decarboxylase was determined by measuring the amount of GABA in MSG using a previously reported modified Berthelot reaction [8]. The reaction mixture consisted of 100 mM MSG, 0.1 mM PLP, 50 μL purified enzyme and 100 μL Na_2_HPO_4_-citric acid buffer (50 mM), pH 5 (unless otherwise stated). The mixtures were incubated at 55 °C for 10 min (unless otherwise stated) and then the reaction was terminated by the addition of 200 μL of borate buffer (pH 8.8). The Berthelot reaction method was used to calculate the concentration of GABA, one unit of enzyme activity (U) was defined as the amount of enzyme required to produce 1 μmol of GABA per minute under the above reaction conditions.

Protein was determined by referring to the Lowry method [21], with bovine serum albumin as the standard. Absorbance was determined at OD_595_ using a spectrophotometer (Thermo Scientific, Waltham, MA, USA).

### 2.6. pH and Temperature Profiles

In order to evaluate the properties of the purified enzyme at different pH, the enzyme solution was mixed with the reaction solution at pH 3–8, and its enzyme activity was measured at different pH reaction systems to determine the optimal pH. To further test the pH stability of the enzyme, the enzyme solution was diluted with the different pH buffers mentioned above, incubated in a water bath for 30 min, and then cooled rapidly at 0 °C to determine the residual activity by standard assay.

The optimal reaction temperature of the purified enzyme was determined by varying the reaction temperature (25–70 °C) under the above standard experimental conditions. To evaluate its thermal stability, the recombinant purified enzyme was reacted at 50 °C, 55 °C and 65 °C for 0–180 min, and the residual enzyme activity was determined. 

### 2.7. Influence of Metal Ions and Coenzyme PLP

The effects of different modulators such as metal ions and chelating agents on the activity of glutamic acid decarboxylase were investigated. The enzyme was incubated with 1 mM of Ca^2+^, Mg^+^, K^+^, Fe^2+^, Fe^3+^, Cu^2+^, Mn^2+^, Zn^2+^, Na^+^, Ag^+^, Al^3+^, Li^2+^, Co^2+^ and EDTA for 30 min, and then the residual activity was determined by standard method. The residual activity of the enzyme and the control (without reagents) was determined by the standard assay.

Enzyme activity was measured at different coenzyme PLP concentrations (0–0.4 mM) to investigate the effect of PLP on LcGAD10s.

### 2.8. Examination of the Ability of LcGAD10s Catalyzed Synthesis of GABA

The ability of LcGAD10s to convert GABA was determined qualitatively by thin-layer chromatography (TLC), and the supernatant of the fermentation broth was spotted onto thin-layer chromatography silica gel plates (Merck) [6]. The TLC spreading agent was a mixture of glacial acetic acid, n-butanol and water (2:4:2, v:v:v), to which 0.4% ninhydrin by mass was added as a color developer and 1 g/L of GABA and MSG standards were used as controls. The chromatographic plate was placed in a chromatographic vat, which was placed in a fume hood for chromatography, and the chromatographic solution was allowed to migrate to about 2 cm from the top of the silica gel plate and then dried naturally. After developing, the plate was completely dried at 115 °C for 5 min and then visible [4].

Various conditions were tested and optimized for the efficient production of GABA by LcGAD10s. Preparation of pure enzyme solution as described above, set up 1 mL of mixed reaction system including 15 g/L MSG, 0.1 mM PLP, 0.4 U purified enzyme and 50 mM Na_2_HPO_4_-citric acid buffer, pH 5, 50 °C (unless otherwise stated), and the yield of GABA was measured by TLC after 2 h of reaction. The effect of reaction conditions on the conversion of GABA was determined by adjusting the amount of enzyme added (0.05–0.8 U), reaction temperature (30–80 °C), pH (3–8), MSG concentration (5–25 g/L) and reaction time (1–6 h).

### 2.9. Application of LcGAD10s in the Production of GABA in Low-Salt Sufu Processing

Tofu is a nutritious and longstanding traditional Chinese ingredient that is mostly made through pulping and coagulation molding from soybeans. Sufu is a typical fermented food made from blocks of tofu through the interaction of a variety of microorganisms and enzymes. Low-salt sufu sample preparation steps and parameters were improved as described by Ma et al. [22]: Fresh tofu obtained from supermarkets was cut into 2.5 cm × 2.5 cm × 2 cm cubes and arranged neatly by leaving space in a glass container. Commercial mold solution was prepared and inoculated onto the surface of the tofu. Pehtzes covered with mucor on the surface were obtained at a temperature of 28 °C, humidity of 90% and incubation time of 48 h. After rubbing, the pehtzes were placed in a closed glass bottle with a dressing of 0.9% NaCl, incubating at 28 °C. After margination for five more days, sufu was extracted from the salt solution.

To examine the level of application of LcGAD10s in fermented low-salt sufu, a supernatant containing GAD (0.5–2 U/mL) was incubated with control (0% GAD) at 30 °C for 1 d. Then, to observe the effect of GABA production by TLC, the amount of GABA conversion was determined by the Berthelot reaction method.

### 2.10. Statistical Analysis Subsection

In this study, three parallel experiments were conducted and averaged for each group of experiments, and the results were processed using Excel 2021 software, expressed as mean ± standard deviation (Mean ± SD), and graphs were made using GraphPad Prism 8 software.

## 3. Results and Discussion

### 3.1. Cloning and Expressing of the Glutamate Decarboxylase Gene from Lactobacillus

**Figure 1:**
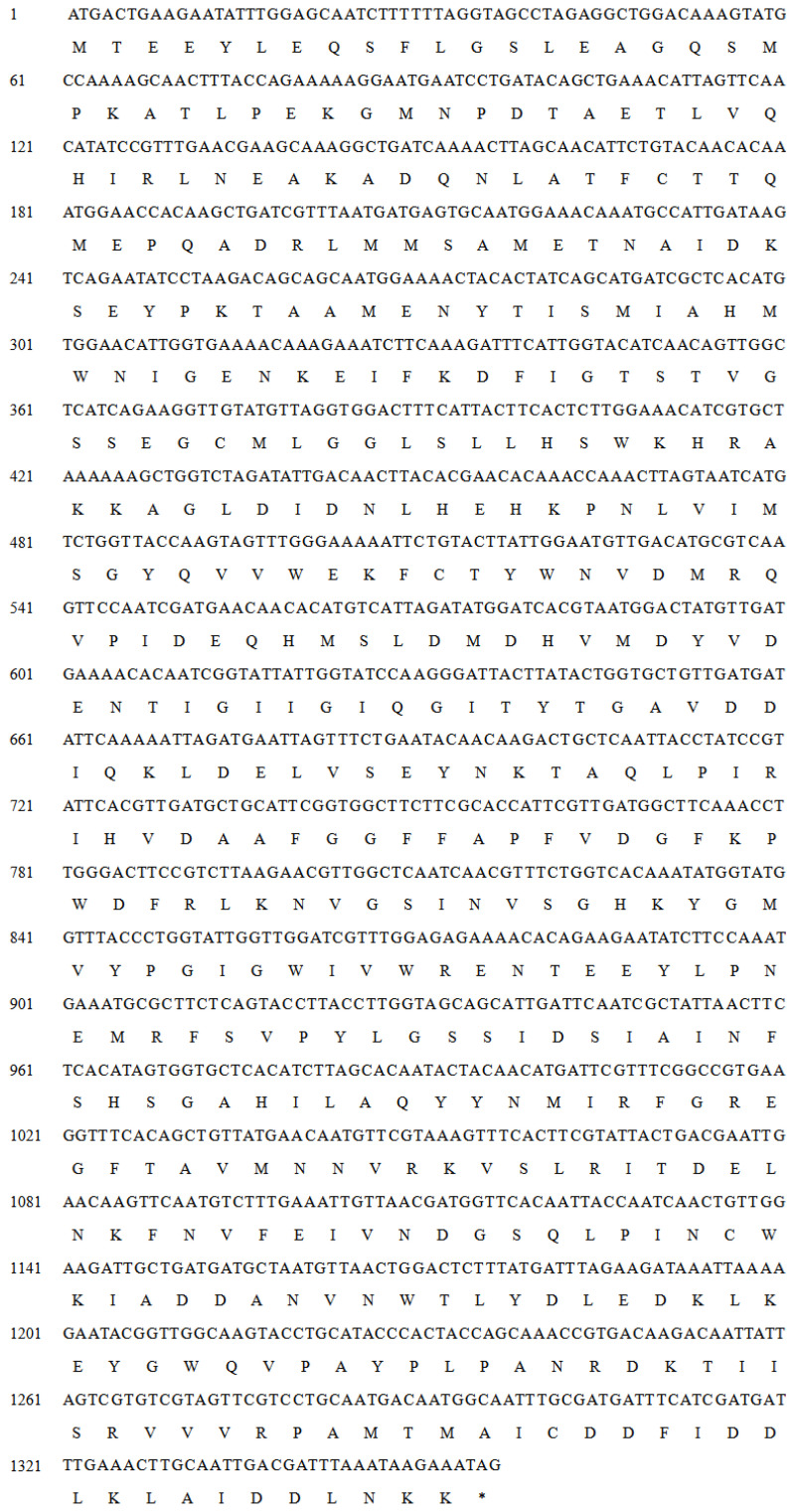
Nucleotide and deduced amino acid sequences of the full-length cDNAs and flanking regions of *LcGAD10s*. * denotes the termination codon.

In order to find an efficient GABA biosynthetic GAD, a genome mining strategy that predicts novel GADs based on reported biosynthetic genes (clusters) was used. In this paper, a set of GADs from *Lactobacillus* were screened from the NCBI database. Here, we were fortunate to obtain a new GAD gene (*LcGAD10s*) from this database and present its nucleotide sequence and the deduced amino acid sequence in Figure 1. The full-length *LcGAD10s* gene has an open reading frame (ORF) of 1356 and encodes 451 amino acids and a stop codon with a predicted molecular mass of 51.2 kDa and an inferred *p*I value of 4.98. A BLAST homology search for *LcGAD10s* amino acids showed terrific novelty, but the clone was never discussed further. *LcGAD10s* had the highest multiple sequence homology with another LAB species *Lacticaseibacillus paracasei* (GenBank No: BAG12190.1) had only 78.7% similarity [23].

The demand for the production of aminobutyric acid has been growing in recent years. Microorganisms present in the natural environment, such as *Yeast* [24], *Lactobacillus* [23], *E. coli* [25] and *Monascus* [26], can be used for the biological synthesis of GABA to synthesize GABA. In particular, LAB has been widely used in the food industry, especially in the production of fermented foods for centuries [12]. For example, as reported by Lyu et al. [27] the *Lactobacillus plantarum* mutant split fermentation could obtain up to 33.52 g/L of GABA, while Wu et al. [28] reported *L. brevis* was a major contributor to the cellular acid resistance. Based on the advantages of LAB-derived GADs, we prioritized the selection of new *Lactobacillus* strains or new enzyme genes to investigate. Additionally, thanks to the progress and low cost of next-generation DNA sequencing technology, a vast amount of genome sequences becomes available. Therefore, we were able to screen and identify the LcGAD10s hypothetical protein quickly in the gene pool, which belongs to the potentially superior GAD proteins, whereupon we successfully cloned and validated the hypothetical protein’s GAD activity and discovered its excellent GABA-producing attributes further. According to the literature research, the enzymatic properties of LcGAD10s and potential application capabilities were demonstrated for the first time in this paper. 

### 3.2. Purification of the LcGAD10s

**Figure 2:**
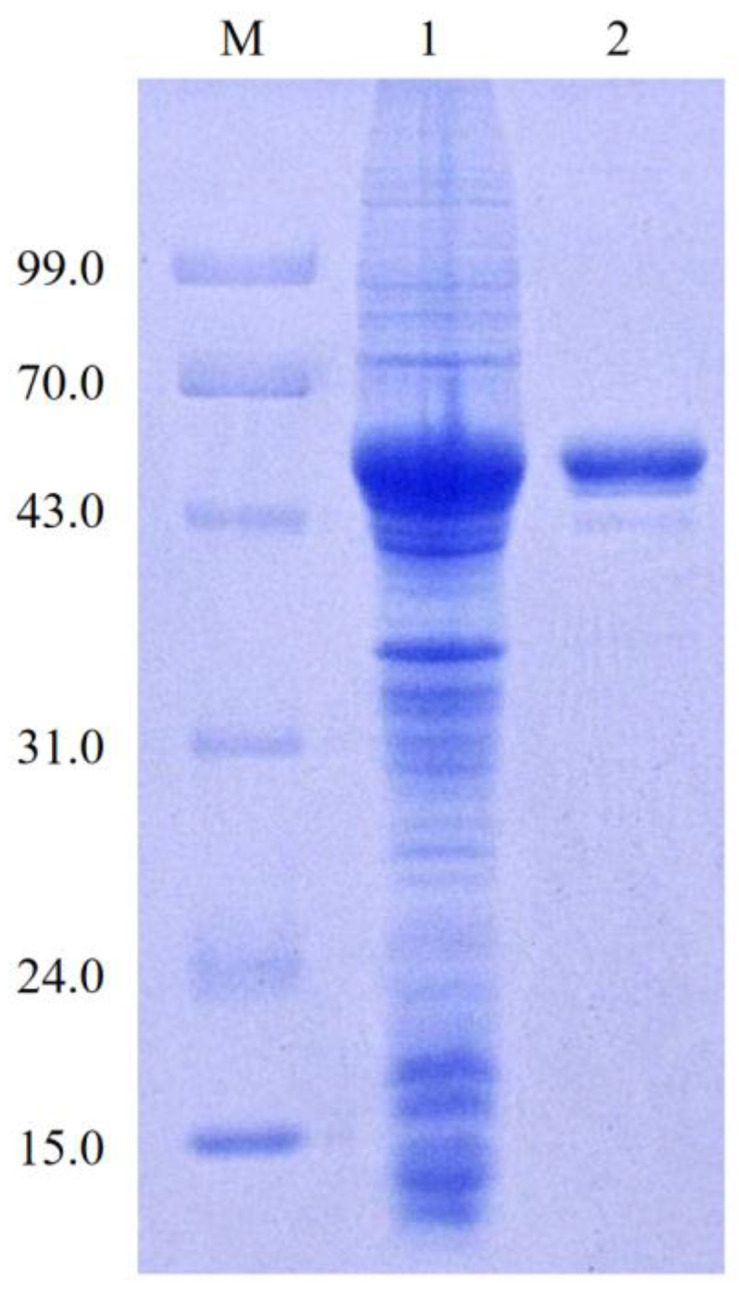
SDS-PAGE analysis of purified LcGAD10s. Lane M, low molecular weight standard protein markers; lane 1, crude glutamate decarboxylase; lane 2, purified LcGAD10s.

LcGAD10s was successfully expressed in *E. coli* and could be purified in one step by Ni-IDA affinity chromatography, the purification profile of which is shown in Table 1. LcGAD10s was purified to 6.1 fold in 41.9% total yield, which specific activity increased from 49.7 U/mg to 303.7 U/mg. The purified LcGAD10s showed a single, homogeneous protein band on SDS-PAGE corresponding to 52.0 kDa (Figure 2), and their sizes were in general agreement with theoretical predictions, indicating that the soluble form of recombinant protein was successfully expressed.

Other microbes reported in the literature had a specific activity of GAD ranging from 0.1–148.0 U/mg [8,29]; however, purified LcGAD10s had a high specific activity, which may have facilitated the conversion of GABA. GAD from LAB is usually composed of identical subunits with molecular masses between 54 and 62 kDa, even when produced heterologous, and forms in mature holomorphs [30]. The reported molecular weight of GADs varies depending on the species and strain as follows: 54.4 kDa for *Lactobacillus brevis* T118 [17], 54 kDa for *L. brevis* IFO 12005 [10], and 53 kDa for *L. zymae* [31]. It is clear that the protein molecular weight size of LcGAD10s is similar to that of typical *Lactobacillus* GAD proteins.

### 3.3. Biochemical Characterization of Purified LcGAD10s

**Figure 3:**
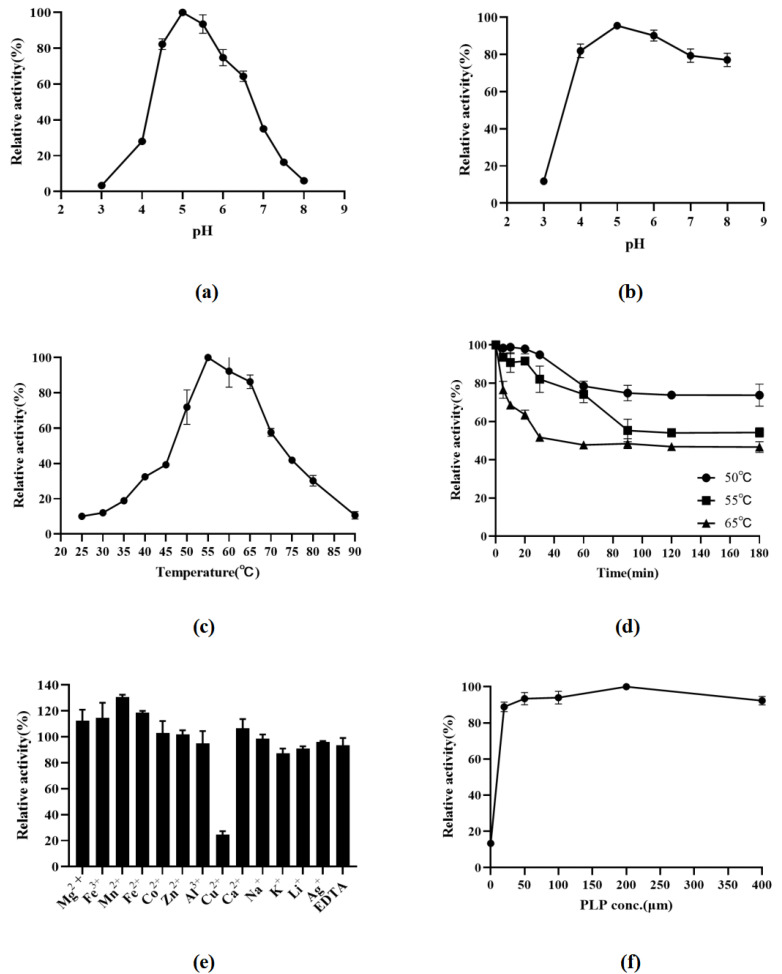
The pH optima (**a**), the pH stability (**b**), the temperature optima (**c**), the temperature stability (**d**), the effects of chemical reagents (**e**), and pyridoxal-5′-phosphate (PLP) concentration (**f**) on the glutamate decarboxylase (GAD) activity. Experiments for each test were conducted in triplicate and reproducible results were obtained.

In this paper, the biochemical properties of LcGAD10s had been characterized for the first time. LcGAD10s exhibited maximal activity at pH 5.5 (Figure 3a). The enzyme exhibited excellent stability, it was stable within the pH 4.5–6 (Figure 3b), and residual enzyme activity can reach more than 80%. The relative activities of LcGAD10s at various temperatures (25–90 °C) were measured under standard assay conditions. As shown in Figure 3c, the enzyme displayed an optimal temperature of 55 °C. And the enzyme was stable up to 50 °C when incubated for 3 h, retaining more than 80% of its initial activity (Figure 3d).

pH is an important factor affecting the catalytic activity of enzymes. Although the properties of GADs varied considerably between LAB species and strains, most LAB GADs exhibited optimal activity at pH 4.0–5.2, such as *Lactobacillus paracasei* (pH 5.0) [23], *L. Brevis* (pH 4–5.2) [4,11], and *Lactobacillus plantarum* (pH 5) [27]. In addition, GADs lost activity significantly at near-neutral pH, the stability of purified GAD over a wide pH range is of great importance for industrial applications.

Temperature affects the conformation of the enzyme protein, the dissociation state of the functional groups involved in the enzymatic reaction, but also the affinity of the enzyme to the substrate, the decomposition of the enzyme–substrate complex, and even the affinity of the enzyme to the activator. The optimum temperature for LcGAD10s is higher than for most genera of LAB (30–50 °C), such as *Lactobacillus fermentum* (40 °C) [15], *Lactobacillus plantarum* (40 °C) [27], and *Lactococcus garvieae* (35 °C) [6]. However, in recent years more heat-tolerant glutamic acid decarboxylases of the genus *Lactobacillus* have also been discovered, such as, for example, *L. Brevis* (55 °C) [4,17] and *L. plantarum* (60 °C) [28]. The results show that LcGAD10s has a high optimum temperature and thermal stability. LcGAD10s was consistent with that reported by Chang et al. [32] where the residual enzyme activity remained above 50% at 60 °C for 3 h. However, there was only 40% residual enzyme activity left at 55 °C for 3 h as reported by Liu et al. [8]. Excellent thermal stability means that glutamic acid decarboxylase is able to maintain its activity at higher temperatures, making it more suitable for a broader industrial application that requires high temperatures to function.

### 3.4. Influence of Metal Ions and PLP

LcGAD10s activity was also evaluated in the presence of each chemical reagent, EDTA and PLP at 55 °C and pH 5.0. From Figure 3e, low concentrations (1 mM) of Cu^2+^ (24%) significantly inhibited the enzymatic activity of LcGAD10s, while K^+^ (87%) and Li^+^ (91%) slightly inhibited the activity. In contrast, Mg^+^ (112%), Fe^2+^ (118%), Fe^3+^ (114%) and Mn^2+^ (130%) could activate the enzymatic activity of LcGAD10s. Other chemical compounds such as Li^2+^, Co^2+^, Zn^2+^, Al^3+^, Ca^2+^, Na^+^ and EDTA had no significant effect on activity. The results in Figure 3f showed that the enzymatic activity of LcGAD10s gradually increased with increasing PLP concentration, with the best activity being achieved at a PLP concentration of 0.2 mM.

Various compounds had previously been reported to be activators of GAD consistent with LcGAD10s, these include CaCl_2_ for *L. brevis* 877G [11], MgCl_2_ for *Lactococcus garvieae* MJF010 [6], and MnCl_2_ for *L. sakei* A156 [17]. Due to the covalent binding of Cu^2+^ to some functional groups in the recombinase molecule, affecting the structure of the active center of the enzyme molecule, such as CuCl_2_ for *Levilactobacillus brevis* HYE1 [4], which strongly inhibited the activity of LcGAD10s. In addition, the same ability to inhibit GAD activity was also consistent with the previously reported KI for *Lactobacillus brevis* 877G [11], and LiCl for *Streptococcus salivarius* ssp. *thermophilus* Y2 [33].

Glutamic acid decarboxylase is a PLP-dependent enzyme that has many PLP coenzyme binding regions, yet intracellular PLP levels are generally so low as to be unable to meet the needs of GAD expression. The addition of PLP had a significant effect on the catalytic performance of glutamic acid decarboxylase [34]. LcGAD10s were present as apoenzyme (apoGAD) without PLP, with the intact GAD-PLP complex (haloGAD) formed by adding PLP and restoring its activity, as in *Enterococcus avium* M5 [35], *Bacillus megaterium* CICC 10055 [8] and *Lactobacillus zymae* [31]. Interestingly, some recombinant GADs showed activity even in the absence of PLP [4]. This suggests that the enzyme may bind to the coenzyme PLP when overexpressed in *E. coli* and is usually present in the form of a GAD-PLP complex [8]. In GABA production, the addition of the coenzyme factor PLP directly affects GABA production, but too low of a PLP concentration tends to have a weak promotion effect on the catalytic activity of GAD, and too high a PLP concentration simultaneously affects the intercellular uptake of substrates and product transport, making it difficult to sustainably improve the catalytic performance of GAD [35].

### 3.5. Examination of the Ability of LcGAD10s Catalyzed Synthesis of GABA

In this study, LcGAD10s was efficiently converted to produce GABA. Among the different reaction factors affecting the rate of GABA yield, which are mostly common and important such as enzyme addition, incubation temperature, initial pH, initial glutamate concentration and incubation time, the fermentation conditions were optimized using single-variable-at-a-time [16].

**Figure 4:**
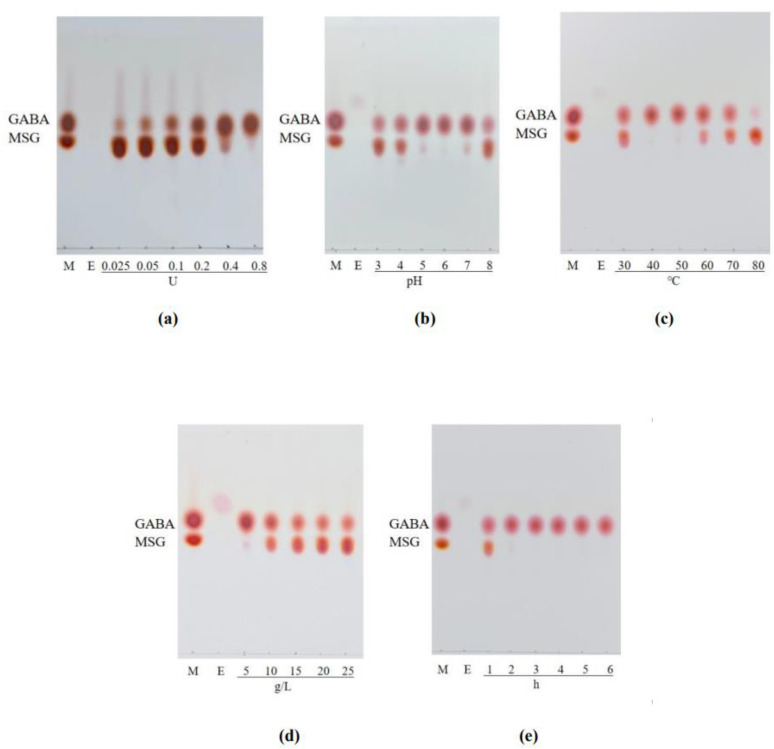
TLC determination of the effect of enzyme addition (**a**), reaction temperature (**b**), pH (**c**), substrate concentration (**d**) and time (**e**) on the amount of GABA production. Lane M: a mixture of GABA and MSG. Lane E: pure enzyme solution.

The effect of different GAD dosages (0.025–0.8 U) on the conversion of GABA by MSG was investigated (Figure 4a). The GABA concentration gradually increased during the addition of 0~0.4 U and then leveled off. When the amount of enzyme added exceeds the critical value, proceeding to increase the amount has fewer effects on the reaction rate [36]. Given that the GABA yield showed no significantly increased with GAD dosage in the presence of 0.8 U of GAD, 0.4 U addition was selected as a reasonable amount of GAD for subsequent experiments.

Figure 4b showed the effect of temperature (30–80 °C) on the conversion of GABA. The results showed enhancement of GABA concentration with increasing the culture temperature from 30 to 50 °C, where maximum GABA produced was obtained, followed by a reduction of GABA production when the culture temperature exceeded 50 °C, which was similar to the optimum temperature of LcGAD10s. Similarly, Tajabadi et al. [16] reported *Lactobacillus plantarum* Taj-Apis362 growth increased with increased temperature and peaked at 37 °C, then decreased over the temperature, indicating that temperature also plays an important role in the activity of the enzyme. Therefore, 50 °C was selected as the center point of extraction temperature in later experiments.

The effect of pH (3–8) on the yield of GABA was investigated. As shown in Figure 4c, the enhancement of GABA concentration with increasing initial pH from 4 to 5, where the maximum GABA production was obtained, followed by a reduction of GABA production when the initial pH exceeded 7. In agreement with our results, GABA production was strongly inhibited at pH 3.0 and 8.0 in Lim et al. [14] report, due to the possibility that high or low pH may lead to partial loss of GAD activity. In addition, most GABA-producing *Lactobacillus* spp. strains are optimized for pH conditions in the range of 4.5–5.5, including the following studies pH 5.31 for *L. plantarum* Taj-Apis362 [16], pH 5 for *Lactobacillus brevis* HYE1 [14], and pH 5.25 for *Lactobacillu paracasei* NFRI7415 [23]. Ultimately, the optimum pH for GABA production by LcGAD10s was determined to be 5.0.

The effect of MSG concentration from 5 to 25 g/L on GABA production was determined. In Figure 4d, during the 1 h of reaction, the effect of different concentrations of MSG on GABA production was significant. The highest GABA production was achieved at 5 g/L MSG and then decreased gradually. Although MSG is an essential substrate for the production of GABA, it was clear that too high a concentration of MSG suppressed GABA production, the optimum is to employ relatively low concentrations of MSG as appropriate, with a previous study reporting for *Lactobacillus brevis* HYE1 [14]. Finally, the optimal MSG concentration was determined to be 5 g/L for GABA production.

As shown in Figure 4e, the reaction was carried out using different lengths of response times (1–6 h). With prolonged reaction time, the yield of GABA significantly increased until 2 h, and then afterward increased slowly up to 6 h of incubation, so a reaction time of 2 h was finally employed. The decrease in GABA biosynthesis could be due to the combined inhibitory effect of high concentrations of GABA and MSG, a similar observation was also reported by Li et al. [37].

### 3.6. Application of LcGAD10s in the Production of GABA in Low-Salt Sufu Processing

**Figure 5:**
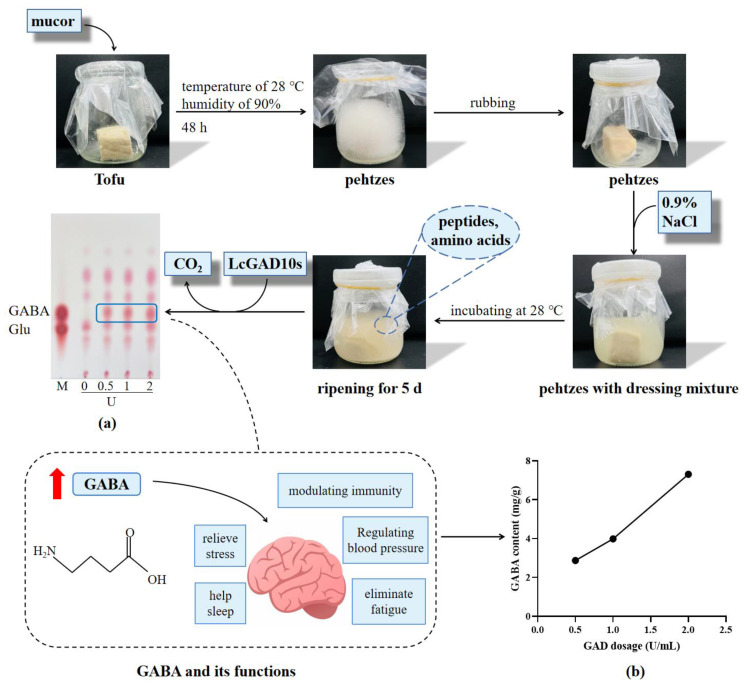
Process flow chart of sufu production, including tofu, pre-fermentation 48 h obtained pehtzes, gross pehtzes after rubbing, pehtzes with 50 mL of stock and post-fermentation sufu. TLC determination of the effect of enzyme addition on sufu (**a**). Lane M: a mixture of GABA and Glu. Determination of transformed GABA content in sufu with different enzyme amounts by Berthelot reaction method (**b**).

The observation of the appearance of the fermentation process of tofu was shown in Figure 5. The concentration of GAD was varied from 0 to 2 U/mL and applied to sufu during a production process. Measurements of fermented sufu with GAD additives are shown in Figure 5a, in which it is obvious that GABA has a rising trend with increasing GAD additions. The GABA content in sufu with 2 U/mL LcGAD10s was determined by Berthlot colorimetric method up to 14.9 mg/g (Figure 5b).

GABA as a bioactive compound has been licensed for use in functional foods in China, the United States, Japan and European countries with demonstrated health benefits. Tofu was chosen to study the GAD application because soybean is a rich source of Glu, which is the GABA-producing substrate for GAD [38]. Due to the effect of microorganisms, the fermentation process of sufu decomposes parts of the macromolecules and produces various amino acids and peptides. Similarly, glutamic acid and lysine are the most abundant among them [39]. Observe the appearance of tofu pre-fermentation for 48 h, the hairy mold grew well. It has tough hair and can form tough bacterial film, which conforms to the requirements of pehtzes production. Various enzymes degrade soy protein resulting in amino acid distribution changes and nutrient content increase [22] when the addition of LcGAD10s allows efficient conversion to GABA of the enriched glutamate. Reported the GABA content of multiple commercial sufu and the average content distribution at 2.8 mg/g of which the highest was 11.6 mg/g [40], and it was also reported that the GABA of a variety of commercially fermented soybean products was determined to be less than 0.4 mg/g [41], which suffices to shows that a great level of GABA can be obtained with our LcGAD10s. This phenomenon is consistent with Park et al. [42] reported significantly higher GABA content of Chungkukjang fermented with the addition of transformed *B. subtilis* (168-GAD) (149.1 μg/g) than that of the control (18.9 μg/g). The increase in GABA content was due to the increase in GAD addition and the decarboxylation of glutamic, similarly, in other reports GAD had been added to foods such as soymilk and breakfast bread to enrich GABA [38].

## 4. Conclusions

In conclusion, this study reports a new glutamate decarboxylase (LcGAD10s) from LAB with a molecular mass of 52.0 kDa and the successful cloning and expression in *E. coli*, and biochemically characterized for the first time, which enriches the resource of high-quality glutamate decarboxylases. LcGAD10s have good heat resistance and other biochemical properties, and still have approximately 80% enzyme activity when held at 50 °C or pH 4–8, indicating better and broader stability for practical application. We also identified several influences such as Mn^+^, Cu^2+^ and PLP that have very significant effects on enzyme activity to support the application of LcGAD10s in different scenarios. In addition, it was verified that LcGAD10s completely converted 15 g/L MSG within 2 h under optimized conditions, it achieved a high capacity to convert GABA. Furthermore, LcGAD10s was verified to be used in fermented tofu products with higher GABA conversion than most commercial sufu and other GADs applications, which provided a theoretical basis for the application of LcGAD10s and its GABA production in food applications and demonstrated great potential for application in fermented GABA-rich foods.

## Figures and Tables

**Table 1:** Purification summary of the LcGAD10s.

Purification Step	Total Activity	Protein	Specific Activity	Purification	Recovery
	(U) ^a^	(mg) ^b^	(Units/mg)	Factor (-Fold)	(%)
crude supernatant	1730.5	34.9	49.7	1.0	100.0
Ni-IDA	725.6	32.0	303.7	6.1	41.9

^a^ Activity was measured in Na_2_HCO_3_-citric acid buffer (50 mM, pH 5) at 55 °C. ^b^ The protein was measured by the Lowry method, using BSA as the standard.

## Data Availability

The data used to support the findings of this study can be made available by the corresponding author upon request.
